# At the Margins of the Medical? Educational Psychology, Child Guidance and Therapy in Provincial England, *c*.1945–74

**DOI:** 10.1093/shm/hkz097

**Published:** 2019-10-17

**Authors:** Andrew Burchell

**Affiliations:** Department of History, Institute of Advanced Study Early Career Fellow and Research Fellow, ‘Cultural History of the NHS’, University of Warwick

**Keywords:** local authority, psychologist/psychology, child guidance, welfare state, children

## Abstract

This article mobilises archival material from local authorities in England to assess the shifting role of psychologists within local school health services from the 1930s through to the reorganisation of the National Health Service (NHS) in 1974. It argues that psychologists were increasingly positioned between therapist, diagnostician and social worker, that this was bound together with a local discourse of children’s emotional well-being and that the increasing fluidity of the psychologist’s role emerged from local policies designed to stress the ‘educational’ nature of their role. In so doing, it extends work by John Stewart on child guidance and more long-standing histories of local, ‘municipal’ medical services. It suggests ways in which the older, localised provision of public health services in Britain persisted after the creation of the NHS and argues the need for a more flexible understanding of what was ‘medical’ about the local welfare state in this period.

Writing in 1981, the British educational psychologist Bill Gillham offered a scathing critique of mid-twentieth-century child guidance. Children’s behaviours were seen too ‘epidemiologically’, with guidance becoming ‘a byword for ineffectuality’ and ‘psycho-analytic explanations’.^1^ ‘It is not surprising’, he wrote, ‘that educational psychologists [such as himself], the only members of the child guidance “team” to go directly into schools … have progressively disentangled themselves from it’.^2^ Gillham was suggesting a narrative that has been articulated, albeit less polemically, by the most recent scholar of the child guidance movement, John Stewart. Adopting a periodisation from the 1920s to the late 1950s, Stewart argues that the movement for team-led and interdisciplinary mental hygiene for children—while undoubtedly a precursor to ‘deinstitutionalisation’ in psychiatric care—was progressively undermined by interprofessional rivalry and competing methodologies, before finally succumbing to personnel shortages.^3^ Underlying Stewart’s narrative is a history concerning the mobilisation of expertise, the desire of mental health professionals to ‘reconstitute’ their professional identities and the changing relationships between the state, voluntary sector and network of local education authorities (LEAs) that administered child guidance.^4^ Through the work of scholars such as Stewart, Rhodri Hayward, Deborah Thom and Mathew Thomson, much more is now known about the development of psychological services—both for adults and children—in twentieth-century Britain, and the ideas behind them.^5^ Yet this is equally a history of local organisation and provides evidence for how local services could operate against (as well as alongside) national trends in psychology and psychological thought.

This article seeks to consider such developments from the perspective of LEAs and educational psychologists, a group related to—and often overlapping in key ways with—the therapists. By mobilising sources from LEAs, and carrying developments in child mental health services beyond Stewart’s 1950s endpoint, I consider how psychologists were reconfiguring their discipline at the local level in mid-century Britain. In analysing through this lens, it is possible to recentre narratives of psychological professionalisation dealt with elsewhere^6^ to examine how psychology was mobilised and promoted by LEA officials within the framework of a localised medical state at a time of increasing centralisation in public health provision and the emergence of more assertive patient groups on the national level.^7^ On the one hand, this is a story of professionalisation and specialisation; what psychologists such as John Hall consider the emergence of clinical and applied psychology.^8^ Yet it also demonstrates the continued valence of mental health sciences to localised social and political projects in Britain. Not only did psychologists persist under LEA control for much longer after the creation of the National Health Service (NHS) in 1948 (and psychiatry’s absorption by it), but LEA psychological provision also became the site for a new series of debates that confronted the NHS’s dominance of health care. These enabled local administrators and practitioners to interrogate the boundaries of ‘the medical’ as a category in mid-century Britain. Should psychology be provided by local authorities or by hospital boards? And what, by extension, should be their role in such sites: a clinical and medically driven one, an auxiliary to a medical service or something more ambiguous and welfare-related? As psychologists, in particular, diversified into sub-fields and branches and as experimental, academic psychology separated from clinical, applied iterations of the discipline, attempts by psychologists in LEA employment to cut across these boundaries are in evidence.

To explore these themes, this article mobilises evidence from several local authorities. Choice of these has been guided by the School Medical Officer (SMO) reports in the National Archives,^9^ as well as sources from central government commenting on relations with local authorities, and, in the final section, the correspondence and submissions of LEAs to the 1968 committee into educational psychology provision led by Arthur Summerfield. These are further supplemented by examining the records of three specific localities: Birmingham (already used by Stewart and an archetypal authority conforming to his narrative of separating functions and decline); Leicester (whose entire municipal medical services have already been the subject of a close study by John Welshman^10^ and whose guidance provision was centred more on psychology); and Brighton (an authority mid-way between the two). Together, these case studies cover two industrial areas and a coastal town whose income was derived from tourism. All of them, however, were autonomous county boroughs, as well as areas with large working-class populations, long histories of municipal activity, intervention and philanthropy. More significantly from a historical point of view, they were also authorities that remained geographically stable over the course of the period 1945–74 (there were few amalgamations with neighbouring boroughs or major boundary alterations).

Such a focus on local tensions and continuity is important. As Welshman notes, at the local scale, with diverse bodies and council committees providing and funding care during the interwar period, it is no simple task to delineate the boundaries of ‘public health’, which often saw school health, hospital care and environmental health overlap. Indeed, even after the creation of the NHS and the wider welfare state, the local remained a site for delivering services and councils did attempt to retain the autonomous provision in some areas.^11^As I shall argue, educational psychologists maintained an ambiguity in their roles throughout the mid-century zenith of the welfare state, with different groups of administrators (local and national) keen to allocate them to specific purposes. Their own voices, meanwhile, sometimes available in annual reports to their employing Education Committees, reveal varied sets of professional practices, which dissolve any sense of professional unity or coherency and instead prioritise the local specifics of negotiating a professional role. In contrast, children and their emotions became central to psychologists in local authority employment, a ‘user’ of the service who could help what, in Chris Nottingham’s formulation for medical social workers, we might term this ‘insecure’ profession to codify its role.^12^

After considering the place of educational psychologists at the beginnings of school health, this article explores their development chronologically across the post-1945 period, culminating in the Summerfield Report and the reorganisation of the NHS in 1974. Central across this periodisation was the ambiguous position of educational psychologists in relation to children and the extent to which, by mobilising discourses of emotionality, they could fulfil multiple roles across medicine, auxiliary medical work and more educational activities.

## Diversity in Local Provision: LEA Child Health Initiatives in the Interwar Period

The richness of the local as a seam of source material and a category of analysis in nineteenth– and twentieth-century British medical history has long been recognised. Work by John Pickstone (Manchester) and John Welshman (Leicester), as well as collaborative work by Stewart, Alyssa Levene and others, testify to the important role of local actors in the shaping and delivery of early-twentieth-century, pre-NHS British health care.^13^ Reflective of British local authorities’ intense decentralisation and autonomy were ambitious—but also highly heterogenous—levels of local authority provision.^14^ While the municipal gospel of progress dates from the nineteenth century, it was ‘energised’, in the words of one collective of scholars, by the growth of support for Labour after 1918 and the rise of exchequer grants to facilitate health and education spending.^15^ (This latter was most certainly the case in Birmingham and Leicester.) The local authority, and especially the municipal county boroughs—towns and cities with a charter and outside the control of the county council—had a key role in the emergence of what George Gosling characterises as the ‘mixed economy of healthcare’ operative during the interwar decades.^16^

Yet many of these characteristically interwar developments in diversifying public health also evidence the increasing siloisation of local health provision, both in terms of which departments or government bodies were responsible for its administration and delivery, and with regard to how these services framed patients and their needs. ‘Maternal and Child Welfare’ provision, for instance, staked very different claims on who should have a right to what kinds of treatment compared to that offered by a Public Health Committee or a School Medical Service (SMS) under the control of an area’s Education Committee. These separate providers could work in concert, and even share personnel in the case of smaller authorities, but were nonetheless protective of their own particular province. This situation by no means ended with the creation of the NHS in 1948, and although many more ambitious local healthcare programmes, such as tuberculosis treatment, were subsumed by the new national service, the SMS continued as separate structures under LEA control until the NHS was reorganised in 1974.^17^

It is worthwhile briefly examining the interwar period here, partly to show how the rhetorical power of the local in British life accounted for the relative timidity of centralisation, even into the late 1940s, but also to outline how Birmingham, Brighton and Leicester arrived at their respective starting points in 1945. One Board (later Ministry) of Education official noted in a memorandum to his superior that efforts to reform the geography of LEAs would lead to entrenched opposition due to what he termed ‘[l]ocal patriotism’: a powerful ‘source of patronage’ in which ‘each cessation of administrative powers whittles away the tradition of local autonomy, of which boroughs and even urban districts are properly proud’.^18^ Child guidance and local psychology were a primary example of these permissive powers, in a context in which the organisation of medical inspection was compulsory for the relevant education authorities (since 1907) but the provision of allied or ancillary services was often the result of the autonomous initiative.^19^ ‘Pride’ in local provision—although difficult to measure historically—is a key component of this.^20^ Such approaches stressed the contemporary importance attached by local authorities to managing their own affairs with a high degree of autonomy, as well as the growing emphasis amongst more radical authorities on providing municipal services under the banner of municipal ‘housekeeping’.^21^ For example, while philanthropic organisations provided vital funds for the initial impetus of child guidance in the late 1920s, as noted by Stewart and Thom, the actual impetus largely came from local authorities and their sense that such a clinic was necessary for the area.^22^

The development of LEA psychology can therefore be inscribed in a broader history of municipal and county government; ones that prioritised the permissive nature of central legislation and saw the state oversee local developments through the control of grant allocations. It is here, however, that questions of nomenclature and claims of originality become difficult to disentangle. The question of where—or who—was the first to provide school psychology and child guidance is complicated by the existence of a patchwork of very different activities at this time, which cannot be easily flattened by retrospective analysis. This is further complicated by the confusing diversity of practices, therapies and ideas, which could circulate under the heading of ‘psychology’ and ‘psychiatry’ in the early twentieth century.^23^ Child guidance and school psychology, where they developed, were often conceptualised as two inter-related services: separate but sharing personnel—which, as we shall see, was the case in 1950s Leicester. In this regard, Stewart’s elision of Leicester in his own work—a more exceptional authority with a powerful psychological component—works against a better understanding of what Deborah Thom views as a more continuous spectrum of types of interwar mental hygiene provision.^24^ This point also works in a further sense, because it is easy to misread Burt’s work for the LCC in the framework of a purely psychometric service; to see his role as that of ‘measuring the mind’ in the words of Adrian Wooldridge’s vast study of twentieth-century educational psychology.^25^ Yet the statistical evidence that Burt amassed fed into research on a range of behaviours in children, including his popular interwar monograph on the psychopathology of juvenile delinquency, much more than the intelligence quotient (IQ).^26^ Indeed, Burt and the psychologists centred on London were responsible for training many of the interwar and immediate post-war personnel in LEA employment. The more Freudian work of Susan Isaacs and the play therapy approaches of Melanie Klein, Margaret Lowenfeld and Kate Friedlander are as much a part of this story as IQ.^27^ While child guidance and psychological services were supposed to have clear divisions of labour between treatment (psychiatry), home visiting (psychiatric social work), leaving psychology with cognitive testing, the reality is that educational psychologists often found themselves absorbing other roles, due to shortages of personnel.

Likewise, LEA psychologists in the provinces had a range of other interests, which do not fit neatly into the notion that psychological services were synonymous with child guidance clinics and structured around a psychometric psychologist, a psychiatric social worker and a psychiatrist interested in behaviour and the family. Thus, for all that John Hall claims that education psychologists constitute ‘the most homogenous group of psychological practitioners’ with ‘broadly similar aims’ in the twentieth century, local factors and appointments could still provide a highly diverse set of practices.^28^ In Leicester, the founder of the school psychological service (as distinct from child guidance clinic) was Raymond Cattell, a psychologist and eugenicist.^29^ In contrast, one of his successors, Agatha Bowley (1909–95; director of the city’s psychological service 1943–48), was more Freudian, publishing widely for a lay audience on childcare.^30^ Yet even Cattell, as a 1964 commemorative volume produced by the Leicester authority was keen to claim, did not wish his role to be subsumed ‘to testing abilities and aptitudes as a psychometrist only’.^31^ These views were echoed by two sympathetic Directors of Education in the city, F. P. Armitage and Elfed Thomas (the latter a psychologist himself)^32^ who were keen to ensure that it offered a ‘consultation service’, as well as ‘therapeutic treatment’ and ‘diagnosis of behaviour problems’.^33^ ‘Consultation’ was an elastic term that covered a range of intervention possibilities for the psychologist. A separate child guidance clinic was established in 1937, and the relationship between the two halves of the structure were regarded by the early 1950s as a particular source of pride.^34^ The hierarchy of control was the crucial demarcation line between the two organs, however; the child guidance clinic ‘directed by the child psychologist … under the supervision of the School Medical Officer’ and the psychological service (also headed by the psychologist) under the control of the education committee and its director.^35^

Leaving aside the accuracy or otherwise of Leicester’s hagiographical attempt to write a history of the service more compatible with its later developments, the ‘psychology’ component of the service was clearly intended to do more from its inception than testing. This is not to suggest that Stewart’s claims about psychiatric dominance in child guidance clinics during the interwar period are incorrect, merely that more thought needs to be paid to the diversity of actors involved. In Birmingham, as Stewart has shown, the service began very much as a psychiatric and therapeutic child guidance clinic, subsequently shifting towards a psychological model only after 1945.^36^ In this case, it was the psychiatrist, Charles Burns, already a figure in child guidance nationally,^37^ who was appointed as ‘medical director’ for the service and oversaw its expansion throughout the 1930s.^38^ Leicester’s service was the diametric opposite of this in being explicitly conceptualised as a psychological one from its inception. Brighton, yet again, was different and complicated by its relationship to East Sussex. Although Hastings, Eastbourne and Hove formed a special joint committee with the county authorities from 1944 in order to establish a common child guidance service (a successor to earlier voluntary clinics in the 1930s) Brighton chose to remain separate and pursue its own system.^39^ Brighton had first proposed a child guidance clinic in December 1939, when it notified the Board of Education of its needs in connection with the reception of evacuees from London and Surrey.^40^ A report by the original ‘Juvenile Care Clinic’ records the employment of a psychiatrist (as medical director), an educational psychologist, a psychiatric social worker and a psychotherapist in 1943.^41^ All of these personnel were on part-time contracts in relation to their Brighton work and were shared with the neighbouring East Sussex joint service.^42^ The area was thus unusual in having a separate psychotherapist (Kate Friedlander, a firm believer in modified Freudian ideas and the importance of the family)^43^ at the moment of its inception, an arrangement which continued for some time.

An over-focus in the historiography on psychiatrists therefore serves to elide the initial early role of the psychologist in the development of ‘school psychological services’ that predate and compete with Stewart’s child guidance clinics.^44^ It also stresses how analysing the local provides a window into individual professional trajectories, which often do not conform to expectations, and how the provinces—out of London and the academic oversight of Burt, Eysenck and others—provided a space for psychological experimentation. The directions of development for each of the three services in Birmingham, Brighton and Leicester were therefore by no means linear and were highly heterogenous. However, all such services were united in their dependency on directly employed staff, something threatened by the arrival of the NHS in 1948. This ensured that, despite local and regional variation, there was a strong degree of national unity in psychologists’ and their administrators’ defence of the uniqueness of the school psychological service (or child guidance clinic) before policy-makers. To do so, however, required presenting the psychologist in specific ways.

## Defending the Link to the Schools: Psychologists in the NHS

Across the late 1940s and 1950s, local authority educational provision underwent several changes. As Brighton demonstrates, the basic architecture of the school medical services was, in many respects, strengthened by the war, and the provision of child guidance and psychological assessment services for children who posed behaviour problems especially so.^45^ In areas where no previous guidance or psychological service had existed, voluntary efforts, working in collaboration with the relevant bodies of local government, further disseminated the practice to LEAs during the period of evacuation.^46^ But these gains were undercut and diminished by the creation of the NHS in 1948.^47^ In the context of child guidance, psychiatric services were split from LEA control, often being shared through the new administrative structure of Regional Hospital Boards (RHBs), with local authorities barely retaining control of psychological testing.^48^ Reorganisation was by no means geographically homogenous, as my examples indicate, but across the services in Birmingham, Brighton and Leicester, it is possible to discern key changes. Firstly, more LEAs made attempts to integrate teachers and schools into the psychological systems, with the result that child psychology came into greater contact with these professions. Paradoxically, this produced a simultaneous need to foreground the educational nature of the service to justify its continuance under LEA control whilst also obliging psychologists to defend their scientific—even medical—expertise.

These post-war developments contributed to the emergence of an increasingly ambiguous, hybrid position for the psychologist within the SMS. The porosity of roles within the post-war landscape of LEA ‘psy’ provision was marked by new concern in central government and attempts by civil servants to enforce common organisational practices across the country. Initially, the newly created Ministry of Education was hostile to psychological control of the services, believing that they should remain medical. But a shift away from this did occur. Circular 347, sent to local authorities in 1959 (in the aftermath of the Underwood Report), began to hint at the desirability of a division between the two; noting that the psychological service was also an ‘essential component’ of child guidance, which should function by ‘advising teachers or parents about learning or behavioural difficulties’ and ‘resolve any underlying emotional troubles’.^49^

As Pickstone notes, psychiatry was not originally included within the development of the NHS, and its later inclusion was often limited, despite pressure from psychiatrists.^50^ Part of the weakness of mental health in the new NHS undoubtedly came from its ambiguous position; both as a healthcare discipline and from an administrative viewpoint. Unlike other areas of medicine, where it was relatively easy to manage the transfer of health functions and capital, the bridge made by the SMS with the LEA and education authorities posed problems for where the division between the two should be drawn. Birmingham’s educational Hygiene Sub-Committee minutes record that the government’s recommendations on the subject of the relations between guidance, psychology and the NHS were felt to be ‘extremely complicated and puzzling’.^51^ The procedure recommended by the Ministry for Education authorities with guidance and psychological services—and that implemented in Birmingham, Brighton and Leicester—was to provide psychologists and psychiatric social workers through the LEA budget and then ‘purchase’ the worktime of a psychiatrist appointed through one of the Regional Hospital Boards.^52^ In practice, however, the question of who should run the service proved highly polemic, with Leicester deciding to invest the service in the School Medical Officer; ‘it being understood’, the Special Services Sub-Committee noted, that ‘the Senior Psychologist and staff, like the other staff of the School Health Service, would ultimately be responsible to the Director of Education’.^53^ It is in this period, as Stewart notes, that the psychologist—as the more permanent member of clinical staff—took over more of the running and coordinating work, in addition to his or her original function liaising with schools.^54^

The Ministry’s cautious ambiguity over the issue can be observed when Alan Moncrieff, honorary secretary of the British Paediatric Association, complained about the exclusion of his profession's from child guidance to the senior medical officer of the Ministry of Education in 1947.^55^ The response, by none other than Underwood, noted that ‘[t]here may be clinics in charge of educational psychologists though I do not know of any, but it is an arrangement which would not be accepted by us at the present time’. But, wrote Underwood, the educational psychologist’s role was vital: ‘through his contacts with schools and his having to deal with educational problems of individual children, [the psychologist] is the main source of reference of cases’.^56^ What is striking here is the fluidity of the service and the professionals within it; where different roles can clearly have different meanings for different groups reliant upon them. The Ministry’s response signals that it viewed the psychologist’s role as a conduit between the two sites of intervention in the form of the school and the clinic. It was this which may lie behind the trend towards increasing psychologisation that has been noted in relation to child guidance by Stewart and indeed prefigures Underwood’s later recommendation in his 1956 report that the psychologist should take on more administrative influence.^57^ Both the medical ‘welfare state’ (as a local network or patchwork of services) breaks down as an object of analysis here, but so does its target—the child or the family—which emerges in a highly abstract, often ungendered, way.

These developments emphasised the increasing movement towards a psychology-centred service and the subtle, but substantial, shift in terminology. In June 1949, the Association of Education Officers wrote to the Ministry seeking a meeting to discuss ‘the lines of development of the Child Guidance service, and in particular, what may be called the schools’ psychological service’.^58^ Indeed, while medical authorities and learned societies, like the British Paediatric Association, were lobbying against the educational psychologist and in favour of psychiatrically trained members of their own professions, local authorities were more concerned about reducing the ‘medical’ aspect of the service and emphasising its educational role in order to retain control over it. The use of the term ‘school psychological service’ in this regard is crucial, since it served to undermine the psychiatrically dominated child guidance and stressed instead the service’s education and school-based rather than medical dimensions. A delegation of several education officers—including Elfed Thomas of Leicester—told the Ministry of their fear that ‘the occasional presence of psychiatrists in Child Guidance Centres would alter the emphasis of the Centre and might mean that the Psychiatrist would get into the position of being in charge of the School Psychological Service’.^59^ Such an approach effectively pit that organisation against the National Association for Mental Health, which had earlier noted its ‘disquiet’ concerning ‘proposals to divide Child Guidance into an educational psychological service based on the schools and a therapeutic service based on the hospitals’. Once again, educational psychologists were singled out for criticism for their lack of ‘clinical experience’, even if, in practice, this may not have been the case.^60^

The division of roles between and within different authorities could also cause tension, with the Ministries of Health and Education both being forced to intervene in a case of ‘deadlock’ between the Sunderland LEA and the Newcastle RHB running since late 1952.^61^ While the Ministry was ambiguous over the loss of the psychiatrist, it nonetheless felt strongly that educational psychologists needed to bridge the gap between school and clinic, and that psychology ‘away from a clinical setting may become sterile and lose its distinctive contribution to mental health’.^62^ This was indicative both of the fluidity of the roles involved in psychological practice and of how important the psychologist’s ability to cross between spaces (school and clinic) was increasingly becoming for administrators and civil servants at the Ministry of Education. It is in this context that the focus on the emotionality of the child, treated in more depth later in this article, should be read: both as a way of appealing to the legal definition of maladjustment and, consequently, as a way of stressing the dual ‘educational’ and ‘medical’ element of the work.

The Ministry’s desire for a psychiatrically dominated service in the early 1950s, however, was rarely imposed by LEAs. One means of encouraging the persistence of the psychologist was by stressing their role in relation to teachers. Three years of experience as a teacher was necessary to qualify as an educational psychologist—theoretically because teachers were expected to respond more enthusiastically to advice from someone who could appreciate their classroom difficulties.^63^ Leicester is one authority where, at least for the post-war period, the efforts to engage teachers and other groups directly with the psychological service has left some archival traces. Agatha Bowley, in one of her psychological reports for the Leicester Special Services sub-committee, felt that the 1944 Education Act had ‘opened up’ new possibilities for the provision of integrated, psychologically aware ‘welfare’ in the education system, singling out initiatives for ‘dull, backward and maladjusted children’.^64^ In June 1945, the Director of Education organised a ‘Brains Trust’ on the role of psychologists, on which Bowley was listed as one of the panellists.^65^ Throughout the 1950s, the service also held annual ‘open weeks’ for teachers, which included lectures on the ‘principles of remedial education’, as well as demonstrations of ‘performance testing’.^66^ Yet while the minute books, and the periodic and annual reports contained therein, reveal that the number of children seen by the psychologists had been increasing steadily—with several fluctuations, between 1945 (631) and its peak in 1957 (842)—the sources of referral were diversifying.^67^ Whereas the 1945 report categorised the ‘majority’ of referrals as coming from ‘Schools in the City’, increasing numbers of young patients were referred directly from parents, family general practitioners, as well as the juvenile courts and probation services (Table 1).^68^

**Table 1:** Sources of referral (as percentages) for the Leicester School Psychological Service, 1949–58

	Schools	Parents	School medical officer	Juvenile court	Children’s officer	Schools branch	Retests	Child guidance	Miscellaneous
1949	50	9	7	6	4	4	9	N/A	11
1950	41	12	8	5	11	8	5	N/A	10
1951	35	8	3	10	17	10	10	4	3
1952	32	5	4	15	12	8	12	11	1
1953	32	5	3	19	10	8	10	10	3
1954	27	6	1	23	10	10	8	8	7
1955	26	4	1	24	7	12	6	15	5
1956	35	6	1	27	5	10	5	9	2
1957	28	6	4	26	7	11	4	12	2
1958	34	4	3	30	5	7	4	7	6

*Source*: LLRRO, 19D59/VII/627/1, At Belvoir House: An Analysis of the Work of the Leicester Schools Psychological Service, 1949 to 1958, City of Leicester Education Committee, November 1959, p. 7.

The response to this by the psychological service was one of alarm. While there is little evidence that schools and teachers were actively turning away from referring cases, the Leicester service was especially keen to foreground the importance of its special, immediate link to the schools and to fight to retain this. The psychologists frequently noted that the majority of interviews with children took place in the schools themselves, rather than a central clinic, actively seeking to de-medicalise this aspect of its activities.^69^ The prospect of a disconnection between the service and the schools was of clearly not insignificant concern to them. In part, this may have been the result of an anxiety that losing the school connection would drive a wedge between the service and the teaching profession. But it may also be a response to the presence of the NHS: a desire to emphasise the distinctively educational role that the service could offer to defend its ‘educational’ authority. Thus, the 1955 psychologist’s report labelled the ‘decline in the number of cases coming from the schools’ a ‘disturbing feature’. ‘[T]he implied loss of contact with the schools’, commented the chief psychologist, Olive Sampson, ‘cannot be accepted with complacency and special attention is being directed to means of righting the situation’.^70^ ‘Contact’ through schools was part of the service’s identity, with the psychological department having ‘built up its reputation’ through school-based cases.^71^

Further evidence of the porous nature of psychological practice can be found in examples of the service’s intention to offer more treatment alongside diagnostics. This created a striking juxtaposition: the increase in discourses of therapy whilst downplaying the medical and reinforcing the school aspects of the psychologist's role. By 1964, a self-congratulatory pamphlet produced by Leicester Education Committee staked the psychologist’s claim to ‘therapeutic knowledge … placed at the disposal of the school’.^72^ In this conception, the psychologist ‘has to combine the skills of the educationist with those of the therapist’.^73^ This meant that spaces increasingly had to be policed. Olive Sampson, who succeeded Bowley as the psychological service’s director in 1948, took issue with the then psychiatrist’s ‘completely undesirable’ practice of using the clinic’s consulting room for seeing ‘adult and child patients in premises where they cannot be segregated’.^74^ Similar views were echoed by the city’s Medical Officer of Health.^75^

There were parallel concerns in Birmingham, although they took a different form. From the late 1940s, Burns was expressing concern in his annual reports—contained within the School Medical Officer’s report—about the increasing case load of the Birmingham clinic.^76^ The Hygiene Committee was equally becoming conscious of the problem of waiting lists, which had begun to grow during the war and had not declined after its end.^77^ It was in this context that Burns made his first plea for a reform of the service and a voluntary diminution in the role of the clinic. Instead, the city needed what he termed ‘a psychological Schools Service, of which the centre but not the whole would be the Clinic itself’, which ‘would have the advantage that more direct touch would be kept with the teachers, and also that many cases would be dealt with without having to reach the Clinic’.^78^ By 1949, seeing ‘more cases for “diagnosis and advice” and take on less for “treatment”’ seemed to have become an officially acknowledged ‘policy’ at the clinic.^79^

While the Birmingham and Leicester services succeeded in mobilising a discourse of proximity to education to defend their position in relation to the area’s school children, a slightly different story emerges in Brighton. While all three were county boroughs—with the same powers as county authorities—Brighton had a more unusual relationship with East Sussex. Together with Hastings, Hove and Eastbourne, it was one a handful of county boroughs centred on the region’s coastal resorts, and, although by far the most populous of the seaside towns, its municipal incorporation in the nineteenth century owed more to its status as a popular destination for royalty than industrial prowess.^80^ It would eventually, in the 1974 local government reform, see its borough status terminated and educational control pass entirely to the county authorities. As we saw in the previous section, the employment of a separate psychotherapist, at least in the initial clinic, may account for the emphasis placed by the long-standing educational psychologist, Dorothy Hammond, on intelligence testing, in what appears (at least at first glance) to be very much a traditional interpretation of her role.

Hammond, according to the census return submitted for the Summerfield Report, had a typical entry into the profession. Having served as a teacher under the LCC between 1929 and 1938, she took a course with the London Child Guidance Training Centre and, prior to that, a correspondence degree (possibly a social science certificate) with the University of London.^81^ Yet even Hammond sought to extend her work beyond the measurement of the mind. She referred in her 1949 report, for example, to the ‘examination and treatment of individual cases of educational or emotional difficulty’ and of her desire to produce ‘some kind of enquiry into the emotional and social condition of the child’.^82^ By 1952, this had become ‘activities designed to help children who were having difficulty in responding to the normal educational, emotional and social requirements of home and school’.^83^ Perhaps the most revealing indication of how her intelligence testing role could support a structuring of the child’s emotions comes in the following extract from her 1952 report:

An individual test of intelligence does more than measure the child’s ability: it provides a setting in which many aspects of the child’s emotional response can be observed. Such qualities as readiness to tackle difficulties, the wish to please, dependence upon encouragement, anxiety, over-confidence and so on are noticeable. One child will boast where another apologies; one will turn silent where another fills the gap in his knowledge with more or less plausible inventions.^84^

Testing was a physical, spatial encounter that facilitated social observation, as well as objective measurement. Indeed, this report also noted her use of ‘projection tests’, such as the Thematic Apperception Test, indicating some desire to measure different aspects of personality, as well as intelligence. With the appointment of a second educational psychologist in 1957, her workload could even be extended to cover administering Rorschach and ‘Family Relations’ tests.^85^ Most of these methodologies were vaguely psychoanalytic in origin, with strong connections to anthropology, foregrounding the importance for Hammond of psychology as an observational practice, rooted in social and clinical contexts.^86^ This was, of course, a departure from the original premise of educational psychology as purely concerned with intelligence or cognitive reasoning and may testify to the endurance of Friedlander’s influence on the service.

The picture that emerges from these case studies hints at the presence and role of diverse practices in the psychological work undertaken as part of the post-war settlement in ‘municipal medicine’. But it equally introduces a cautious ambiguity around where these fit as ‘medical’ (or ‘educational’) practices. Psychologists in all three authorities constructed themselves as agents capable of moving between the setting of the clinic and the school, between and beyond a straightforward ‘medical’ role, even if this was by no means totally accepted. Even Leicester’s calm pride in its service may have belied a system of ‘vested interests’ and resistance to reform by the 1950s, as Welshman has suggested.^87^ Psychology was not always successful in positioning itself as a social-medicine counterpart to state medicine, as the next section argues. Yet what does emerge most forcefully in this consideration of Brighton, Birmingham and Leicester is not only the shift in power towards the psychologist identified by Stewart but also equally something with which few scholars of this topic have engaged explicitly: the shifting nomenclature away from the child guidance model towards a ‘school psychological service’. This name both foregrounded the educational qualities and the psychological aspects, to the detriment of the teamwork approach of the interwar period, but also highlighted further ambiguities surrounding the service’s medical claims.

## Emotions, Therapy and Engaging with Children

The question posed by this equivocation is what, precisely, was envisaged as ‘medical’ in these services; not only for the psychologist to stake a claim on some medical activity but also to position themselves as still sufficiently educational to retain autonomy from the NHS? One way, I wish to briefly examine, is in how they thought about their role in relation to treating the child’s emotions. Some evidence of this can be seen in Hammond’s attempts to measure personality and feelings in her Brighton work, yet it could also involve collapsing boundaries of diagnosis and treatment. As even Burns—a trenchant critic of psychological dabbling in psychotherapy—noted in his report for 1950, psychological ‘diagnosis’ could itself be therapeutic: ‘it is necessary to be content with “diagnosis” in many cases. This does in fact amount to preliminary “treatment”, since the problem is explored, advice given and some of the tension relaxed’.^88^ The submission from Brighton’s Director of Education, W. G. Stone, to the Summerfield committee in 1965 also stressed this point, noting that Hammond was ‘particularly interested in therapeutic work’ but had ‘asked if she could be relieved of clinical [i.e: child guidance] responsibilities’.^89^ For Stone, as for his equivalents in Birmingham, the school psychological service needed to act as a filter, ‘referring only a few [cases] to the Child Guidance Clinic’. Moreover, it was inconceivable ‘that the medical element should be dominant in a field where the main weight, in my view, should be given to educational, psychological and social factors, rather than to medical ones’; the ‘other professional workers’ in the clinic (which presumably included the psychiatric social workers and the psychiatrists) should simply be ‘consultants as required’.^90^ The perception that psychiatry was in some sense distant from the actual world of the child—whereas psychology was not—seems to have been largely shared by his colleagues (although we can speculate that labour shortages and an unwillingness of psychiatrists to engage in school-based work may well have played a role).

The type of ‘therapy’ that psychologists could offer children was rarely specified directly in this material; although, where the author did mention details, it was often related to an ability to engage with children through play. Play (as a component of behaviour) occupied a key role in the clinic’s diagnostic and therapeutic work. As we have seen, the initial forerunner of the East Sussex clinic employed Kate Friedlander as a therapist, but this does not mean that play therapy was absent in other cases. For Burns, play was simultaneously diagnostic and therapeutic. It revealed not only the child’s ‘inner life’ but also equally ‘has such curative value for children, giving them an outlet for their interests and creative abilities’.^91^ It is worth dwelling on the play aspect as evidence of individual idiosyncrasy and local heterogeneity within the services emerging at this time. Although the first full-time psychologist to the Birmingham clinic was not appointed until 1938, one of her roles from her arrival included the organisation of ‘the playroom’, indicating the delegation of this form of therapy to psychologists.^92^ The initial nominee to this post clashed with Burns soon after her appointment—the latter accusing her of having no ‘originality of outlook with regard to the principles and methods of play therapy’—and she subsequently left the service to pursue further training.^93^ In Leicester, where the psychologist was more powerful, play therapy was also being undertaken. Among the requisitions made when the service moved to its home in the city centre in the late 1940s were furnishings for ‘play rooms’, including the construction of a ‘play house’ and the fitting of a ‘zinc tray with cold water for the children’s use’.^94^ ‘Play therapy’ was described in one of the service’s annual reports as ‘the best means of assisting the child to come to terms with himself’, and mention was also made of ‘rhythmic therapy classes’, which were considered ‘beneficial both to the inhibited, nervous child and to the naughty, unruly child’.^95^ As a Leicester pamphlet acknowledged, ‘it is interesting to see how the function of the psychologist in a mature service has developed from the “classical” role at first assigned him’.^96^ Thus, play therapy, as a window onto what Stewart as called the child’s ‘emotional landscape’, was tacitly accepted as part of the psychologist’s remit but could be policed by superiors.^97^

Despite the impression offered by the above case studies, it would be wrong to suggest that the psychologist was universally accepted within the school health service and by local educational administrators. Analysing LEAs in which the psychologist’s role was diminished indicates how the ‘emotional’ appeals of the psychologists could equally fall into the interests of other bodies or professions. Such groups could mobilise children's emotions to shut down access to both psychological and psychiatric models of child behaviour and militate against their inclusion in medicalised services under education committees.

For example, the Midland borough of Dudley had no effective child guidance or school psychological services until 1960, when it appointed an educational psychologist ‘for the first time’. Prior to this, the area’s Chief Education Officer claimed, it had access to an NHS psychiatrist and a nurse occupying the functions of a ‘psychiatric social worker’.^98^ To see the former required a trip of 25 kilometres to the Worcestershire town of Bromsgrove.^99^ The absence of a service here, and the concerns which animated it elsewhere, may be due to the industrial and deprived nature of the area, which saw elected and appointed local officials place more emphasis on eliminating the physical diseases of poverty, like tuberculosis and rickets, which were still common into the 1950s.^100^ When a service of sorts was finally established in 1956 (albeit without a psychologist), the majority of diagnoses and referrals in the area were for anxiety disorders rather than behaviour; comprising exactly a quarter of referrals in 1956 and rising to exactly a third in 1958.^101^ This suggests that emotional conditions were suddenly rendered more visible now that the restraint on the service had been removed.

Dudley’s neighbouring borough of Smethwick also had difficulty in establishing a child guidance or psychology service, but the attitude of the school medical officer seemed to indicate entrenched resistance to the principles underlying guidance. This is all the more strange, as Smethwick’s Education Committee had been debating the issue of a child guidance clinic since at least the late 1920s, when they began correspondence with the national Child Guidance Council.^102^ As late as 1960, when it engaged in correspondence with the Ministry of Education on the issue, the borough’s Chief Education Officer noted that ‘a Child Guidance Service is an essential feature of the service which the local education authority ought to provide’ but felt that ‘a serious shortage of properly qualified staff’, as other authorities found to their cost, would hinder development.^103^ While economics and practical staffing issues dominated this letter, this was not the case in discussions of child guidance clinics and educational psychologists by the LEA’s medical officer. Richard Dodds, the area’s Principal School Medical Officer, wrote in his 1957 report that there was ‘no child guidance clinic in Smethwick’, and that he was ‘as yet not wholly convinced that the mental health and educational progress of our children are suffering greatly from this lack of psychiatric guidance’, although ‘a time may well come’ when it could prove necessary.^104^

Whether Dodds’ views here are all that they appear at first glance is not clear, and they may well indicate a more profound resistance on his part to the establishment of such a clinic. This is all the more so, given that 2 years later, his report included another cryptic reference to the possible future establishment of a service. Directly referencing his comments in the 1957 report, he noted that:

The passage of time has confirmed the view that a full scale child guidance service can be invaluable to a minority of the more difficult maladjusted children and beneficial to a larger number of such children. I am, however, unchanged in my opinion that the experienced school medical officer can and does provide a great deal of practical and down to earth help for the emotional problems of the maladjusted child.^105^

What appears to have been at issue here was really the role of the school health service in relation to dealing with ‘emotional’ difficulties; a concern not so much with protecting the distinctive nature of the ‘school’ service against the encroachment of the NHS and external agencies as negotiating between hostile medical professions. Dodds’ obituary in the *BMJ* suggests that he was a proponent of community health and health education, and perhaps an interest of this nature would predispose his attitude towards psychology.^106^ As in Brighton, emotionality appears to have been of particular rhetorical importance in carving out a position for professionals working with the mental health of children, albeit here in the service of medical officers as opposed to psychologists. Like Dudley, Smethwick enjoyed a productive relationship with neighbouring authorities in this regard but appears to have been forced to send cases outside of the borough to a clinic in Solihull (on the opposite side of Birmingham) for treatment. In the 1948 municipal year, only two children benefited from this.^107^ It seems that children’s emotions were a polyvalent discourse within the professional context: amenable for a variety of motives and both for and against the psychologist’s position.^108^

## The Summerfield Report and Its Aftermath

How the local issues played out at the national and institutional level is the concern of the final section of this article. Specifically, it examines how anxieties about psychological provision were seen through the prisms of health and local government reform in the aftermath of the Summerfield Report, published in 1968 as *Psychologists in the Education Services*.^109^ Professor Arthur Summerfield, of Birkbeck College, was commissioned to chair the committee—which also included representatives of educational psychology and local authorities, including Leicester’s Elfed Thomas and Olive Sampson—in response to concerns within the Department for Education and Science (the successor the Ministry of Education) over the supply of educational psychologists for local authority services.^110^ It followed on from earlier reports, including the Underwood Report (which made specific recommendations not only concerning the organisation of child guidance clinics but also the staffing levels within them), and the Newsom and Plowden Reports, which advocated greater attention to the emotional, psychological and sociological make-up of children within educational policy.^111^ Analysis of the submissions received provides a useful window into the complexities of educational psychology at the time, as well as the competing roles it was expected to play in the eyes of senior administrators and Chief Education Officers, at least in the mid-1960s. These corroborate the general trend, identified above, for the psychological services to be maintained separately from their child guidance counterpart, with a specific departmental empire building structure determined to preserve their educational nature.

Thus, in Cambridgeshire and the Isle of Ely LEA, child guidance was firmly placed under the RHB, while the ‘Schools Psychological Service is administered as part of the advisory machinery of the LEA, [and] the educational psychologist is considered as a member of the advisory staff’.^112^ Once again, ‘emotional behaviour’ was mobilised alongside ‘educational problems’ to argue for this configuration of the service.^113^ In contrast, in other, predominantly rural counties—including Herefordshire and Rutland—child guidance did not exist at all, instead provided entirely through hospitals and neighbouring authorities. Educational psychologists were nonetheless employed by these authorities, indicating a complete rupture between the educational and medical aspects of the role. In the case of the latter, the psychologist took on the role of a ‘psychotherapist to the Child Guidance Clinics’ being described as a ‘general purpose child psychologist’.^114^ The precise nature of this therapeutic work was not defined, but it would not be unreasonable to assume, from the evidence in Birmingham, Brighton and Leicester that it included play therapy, as well as remedial teaching. The appeal to emotionality was further exemplified in the submission from the West Riding of Yorkshire, where a letter from the LEA’s notoriously progressive Chief Education Officer, Alec Clegg, outlined ambitious plans to use educational psychologists to ‘encourage the development of a primary school environment, rich in sensory experiences, and thus proselytise the preventive role of the school in the development of behaviour and emotional difficulties’.^115^ Once again, behaviour and emotionality were understood as central to the psychologist’s role.

Chief Education Officers, it appeared, had a particular understanding of where the psychologist should stand in relation to the child guidance service; especially as the psychiatrists were often under the control of the RHB and not the local authority. The East Sussex LEA, for example, placed the teacher and parent at the centre of its psychological service, particularly providing ‘help and advice’ with ‘children whose failure to learn or whose emotional development or behaviour causes concern may be in need of investigation’.^116^ Yet, crucially, the ‘psychologist is dependent upon the teachers to pick out children in difficulty’.^117^ An extract from the school psychological service’s reports from Wiltshire described the function of the service as dealing with three main types of problem, including ‘asocial or anti-social behaviour in or out of school’.^118^ For local administrators—and perhaps the psychologists for whom they were ventriloquising^119^—the service was very much educational in scope, and the treatment of behaviour and difficulties defined under the diffuse heading of ‘emotional’ were important by the mid-1960s in justifying the continued need for such a service.

Indeed, one of the Summerfield committee’s main findings was the extent to which psychologists were already overextending their disciplinary boundaries and providing a variety of different quasi-medical functions in relation to children’s mental health, and particularly in relation to emotional and behavioural difficulties. This was most visible in the evidence and recommendations regarding training. According to civil servants briefing the publications department ahead of the appearance of the report, Arthur Summerfield was keen ‘to avoid any suggestion that intelligence testing by routine methods is a substantial part of educational psychologists [*sic*] work’, and this even affected choices for the design of the report’s cover.^120^ The Child Guidance Training Centre, in a summary submitted to Summerfield, noted that its syllabus included both work on ‘child development’ (‘the putting together, as far as possible, of current knowledge of intellectual and emotional development with what is known of the patterns of physical development’) but equally ‘in relation to emotional stress and maladjustment, the aims and methods of psychotherapy are discussed’. Thus, while the centre ‘make no attempt to equip the psychologist to be a “Rorschachist” … we try to give him enough insight and interest to inspire him … to take a formal long course in this technique’.^121^ Although the document claimed that ‘no attempt is made to equip psychologists to undertake individual psychotherapy’, it is hard not to interpret the tentative wording of this syllabus as tacitly condoning the direct opposite.^122^ The equivalent course at the more psychoanalytic Tavistock (an institution that may come as a surprise as a training ground for psychologists) regarded the therapeutic role as outside the scope of psychologists’ training. Nevertheless, and with perhaps deliberate ambiguity, they also noted that these functions—and especially play therapy—should ‘in the future … be seen as one small band in the spectrum of preventive and therapeutic action which … will be mainly the psychologist’s province’. It likewise criticised the assumption that the psychiatrist alone was uniquely equipped or trained to carry out these methods.^123^

Although beset by an initial delay in publication, the Summerfield Report finally appeared in October 1968 and teaching unions and other organisations immediately offered their views on its key findings.^124^ However, two key developments had problematised the landscape of local authority provision since the committee had been given its initial remit. The first, the Seebohm Report, published more or less contemporaneously with Summerfield, had recommended the transfer of child guidance functions to its proposed social services departments (a prime example, perhaps, of how, by the mid-1960s, applied social science was vying with psychology to be the unifying force of the welfare state).^125^ The second, emerging in the early 1970s, concerned the reorganisation of the NHS, which threatened to reignite the debate over where control of mental health services should be.

For the civil servants, aware of the dangers of navigating two reports with quite different recommendations, the complexities of defining the ‘medical’ issue were the primary obstacle to implementation.^126^ By 1971, civil servants at the Department of Education and Science were advising that ‘local education authorities must not only continue to have an educational psychological service but should also retain power to provide child guidance if necessary, concurrently with the National Health Service’. This was despite an acknowledgement that each one had ‘always been something of a joker in the pack and rests on no specific statutory power’.^127^ Civil servants saw a need to compromise: with child guidance easily dissoluble into the NHS but the preservation of independent psychological services a red line in the negotiations.^128^ It was here that their lack of clarity over psychology was thrown into focus, with one civil servant noting that ‘I find it difficult to grasp clearly the actual physical shape of the school health service. It does not have much property and seems mainly to comprise a wide variety of men and women.’ As he enquired in an attempt to flow chart and map the structure of the school psychological service: ‘in what sense is it a service?’^129^

He may equally have asked in what sense it was ‘medical’. One legal advisor noted that his doubts over the Department’s stance were ‘partly I suppose because of my ignorance of the functions of educational psychologists’, asking ‘[d]o not psychologists also provide something which in law would be regarded as treatment?’^130^ These legal uncertainties reflect the broader professional ones at the centre of this article. The inability for civil servants to answer such questions not only testifies to changes within the services but also to their diversity and to the apparent interchangeability of the terms ‘child guidance’ and ‘school psychological service’—ambiguities that LEAs had themselves promoted from the 1950s onwards. Ultimately, the aim of the Department was to preserve, as far as possible, the powers of LEAs over the provision of psychology services, and child guidance provision could be sacrificed within this because, as the minutes of a meeting between the two departments agreed, the ‘three elements constituting the existing service seemed likely to evolve most appropriately as distinct – though closely related – services’ and thus ‘seemed the responsibility of the health, education and social work services respectively’.^131^ It suggested, in other words, that the sustaining team approach was no longer relevant, and that each separate division—social, medical and educational—should now respond to its own needs.

## Conclusion

As the aftermath of the Summerfield Report makes clear, psychologists could ultimately no longer sustain nor monopolise the discussion and treatment of the child’s emotions. Or, alternatively, as in the case of Smethwick, they had failed to successfully fix the idea of the emotions as something within psychology’s remit. At the same time, by the 1970s, there was open acknowledgement among civil servants that the role of the psychologist had greatly expanded. Exploring the complexities of the construction of LEA psychology and psychiatry—and their competing forms of expertise—demonstrates the porosity of these professions at a local level and hints at ways in which schoolchildren’s mental health was a key battleground for a host of actors in twentieth-century Britain to develop political and professional authority. In this, the question of how far the service was ‘medical’ as opposed to ‘educational’ became paramount. In much of the historico-sociological literature on professionalisation of medicine, the emphasis is often placed on general practitioners and specialists. In the American– or French-centred narratives, these are often told through a narrative of state recognition for corporate identity and professional independence within a free market.^132^ The problem with this is twofold. Firstly, such analyses often concentrate on the central state as a legitimating agent and elide the local. Secondly, even if doctors and psychiatrists were autonomous, self-regulating professions (as they still are, theoretically, within the NHS), not all professions grouped under the aegis of the ‘medical’ were. Such groups could still be employed directly in various areas of localised expertise: specialists not in terms of knowledge but of localities and specific patient groups (children in local schools). More importantly, it completely elides other medical professions—such as nurses—who were brought into the parameters of the state, nor the psychologists examined here who remained in *local* employment but increasingly saw their position in relation to other forms of medical authority fluctuate. More work is needed on those at the margins of these processes.

School psychological services were in fact doubly marginalised within the ‘medical’. On an administrative level, by virtue of being under the control of education departments rather than public health committees, their medical status was always in dialogue with their ‘educational’ nature. On a more abstract level, as a consequence of diverse practices among psychologists (such as intelligence testing and play therapy), they were at the margins of ‘the medical’ itself; somewhere between diagnostic auxiliaries and therapeutic providers. Psychologists and their LEA superiors seem to have deliberately instrumentalised concerns around the emotional life of the child as a way of influencing policy and advancing the position of their profession. In most areas, psychologists transgressed professional boundaries; they did not need to ‘disentangle’ themselves (to quote Gillham’s formulation) from their psychiatrist colleagues, they were already independent. Yet I wish to suggest, against Adrian Wooldridge’s focus on academic psychology, and in an extension of Stewart’s examination of voluntary sector provision, that the local authority level was the key site at which educational psychology and its relation, child guidance, were developing in the twentieth century. It is the site where its porous nature, in opposition to attempts to impose an academic and professional straightjacket, was most visible. LEAs went from the role of instigator and pioneer of treatment to that of subservient authority, desperate to retain some level of local control over these services. Indeed, the very fact that these services were operating under LEAs and the government body responsible for *education*—as opposed to health—is not insignificant.

But the local, provincial services were equally the prominent location in which the tensions inherent in the construction of psychology and psychiatry as ‘medical’ professions (or otherwise) emerge most visibly. This is especially so if we consider the shifting power relations between centre and province that the cases explored in this article illustrate. Such analyses provide a window for reflecting on the porosity of ‘the medical’ as a category of analysis within mental health care. Twentieth-century Britain witnessed the blurring of several boundaries in health care, under the impetus of state-driven health consumerism, education initiatives, as well as social medicine and a Freudian-influenced mid-century psychological consensus examined here.^133^ Yet the other side to this story is one of local and national politics: of decentralised local government agents seeking to redefine psychology, as well as efforts to impose uniformity from above on what the Ministry of Education and its successors rapidly realised was a complex and varied web of provision in which traditional authority could be inverted. Government departments and LEA committees were keen to preserve their autonomy and their powers, and this allowed them scope to redefine treatments to suit their own agendas or tolerate psychologists’ meddling in the medical domain. At this local level, efforts to see the welfare state as a monolithic, coherent entity dissolve under the heterogeneity of a profession like the psychologists. Sources from these groups betray a high degree of unease with defining where the parameters of psychology ended and where medicalised psychiatry began. For what were the ‘emotions’ acting as a surrogate in these cases? Or, to put it another way, what was the wider socio-cultural value and valence of the child’s emotions within medical expertise at this time? In attempting to answer these questions, a more complex, even messy, ordering of psychological and medical expertise within post-war Britain than has been acknowledged by historians, or even by contemporaries like Gillham, emerges. We should therefore be cautious of assuming that, because child guidance was waning by the 1950s as Stewart ably demonstrates, psychology’s position and role in ‘municipal medicine’ went into decline with it. Instead, educational psychology’s post-war porousness reveals much about how more long-standing iterations of ‘municipal medicine’ were able to resist NHS encroachment into the territory of children’s well-being and health. 

## Footnotes

1 Bill Gillham, ‘Introduction’, in Bill Gillham, ed., *Problem Behaviour in the Secondary School: A Systems Approach* (London: Croom Helm, 1981), 11–12.

2
*Ibid*., 12.

3 John Stewart, *Child Guidance in Britain, 1918–1955: The Dangerous Age of Childhood* (London: Pickering & Chatto, 2013); and John Stewart, ‘Child Guidance and Deinstitutionalisation in Post-war Britain’, in Despo Kritsotaki, Vicky Long and Matthew Smith, eds, *Deinstitutionalisation and After: Post-war Psychiatry in the Western World* (Basingstoke: Palgrave Macmillan, 2016), 175–93.

4 Stewart, ‘Child Guidance and Deinstitutionalisation’, 176.

5 Stewart, *Child Guidance*; Rhodri Hayward, *The Transformation of the Psyche in British Primary Care, 1870–1970* (London: Bloomsbury, 2014); Mathew Thomson, *Psychological Subjects: Identity, Culture, and Health in Twentieth-Century Britain* (Oxford: OUP, 2006), 110–17; Nikolas Rose, *Governing the Soul: The Shaping of the Private Self*, 2nd edn (London: Free Association, 1989), 162–204; Deborah Thom, ‘Wishes, Anxiety, Play, and Gestures’, in Roger Cooter, ed., *In the Name of the Child: Health and Welfare, 1880–1940* (Abingdon: Routledge, 1992), 200–19; Adrian Wooldridge, *Measuring the Mind: Education and Psychology in England, c.1860–c.1990* (Cambridge: Cambridge University Press, 1994).

6 Stewart, *Child Guidance*, 147–49.

7 Alex Mold, *Making the Patient Consumer: Patient Organisations and Health Consumerism in Britain* (Manchester: Manchester University Press, 2015).

8 John Hall, ‘The Emergence of Clinical Psychology in Britain from 1943 to 1952 Part I: Core Tasks and the Professionalisation Process’, *History & Philosophy of Psychology*, 2007, 9, 35.

9 See The National Archives, Kew (TNA), series ED 137.

10 John Welshman, *Municipal Medicine: Public Health in Twentieth-Century Britain* (Oxford: Peter Lang, 2000).

11
*Ibid*., 17.

12 Nottingham stresses the liminality of these professions and their need to organise in order to resist external impositions: Chris Nottingham, ‘The Rise of the Insecure Professionals’, *International Review of Social History*, 2007, 52, 446–47. While not wishing to rehearse already well-known works within these debates, there are myriad interpretational frameworks circulating for disentangling the place of ‘expertise’ in twentieth-century Britain. Harold Perkin suggested a rise-and-fall framework for the role of the expert; coming to prominence in the immediate post-war era of welfare consensus, only to suffer a sustained attack by the twin forces of neo-liberalism and anti-authoritarian, anti-psychiatric radicalism after the 1960s [Harold Perkin, *The Rise of Professional Society: England Since 1880* (London: Routledge, 1990), 475–83]. The sociologist Mike Savage similarly described the rise of a technocratic class in the mid-century. Focusing on the shifting role of the social scientists and the allied social research professionals, he presents a form of ‘technical identity’ based on direct involvement in the gathering and interpretation of data, which challenged and eventually superseded an older, ‘gentlemanly’ approach based in the traditional university structure [Mike Savage, *Identities and Social Change in Britain Since 1940: The Politics of Method* (Oxford: OUP, 2010), 67–111].

13 John Pickstone, *Medicine and Industrial Society: A History of Hospital Development in Manchester and Its Region, 1752–1946* (Manchester: Manchester University Press, 1985); Welshman, *Municipal Medicine*; Alyssa Levene *et al.*, *Cradle to Grave: Municipal Medicine in Interwar England and Wales* (Bern: Peter Lang, 2011).

14 Robert J. Morris and Richard H. Trainor, eds, *Urban Governance: Britain and Beyond Since 1750* (Aldershot: Ashgate, 2000); Robert Colls and Richard Rodger, eds, *Cities of Ideas: Civil Society and Urban Governance in Britain, 1800–2000. Essays in Honour of David Reeder* (Aldershot: Ashgate, 2004).

15 Levene *et al.*, *Cradle to Grave*, 86–87; Welshman, *Municipal Medicine*, 27; Thom, ‘Wishes, Anxieties, Play, and Gestures’, 214. See also Tim Willis, ‘Politics, Ideology and the Governance of Health Care in Sheffield Before the NHS’, in Morris and Trainor, eds, *Urban Governance*, 128–49. For the case of Leicester especially, see Welshman, *Municipal Medicine*, 70.

16 George Gosling, *Payment and Philanthropy in British Healthcare, 1918–1948* (Manchester: Manchester University Press), 4.

17 Bernard Harris, *The Health of the Schoolchild: a History of the School Medical Service in England and Wales* (Milton Keynes and Philadelphia: Open University Press, 1995). For ‘territorial debates’ between different government departments, see: Stewart, *Child Guidance*, 137.

18 TNA, ED 136/131, ‘Note on Chapter IX of the Spens Report’, Howlett [?], Davidson and Williams to the President, n.d. [*c*.1938], 15–16.

19 Harris, *The Health of the School Child*; Welshman, *Municipal Medicine*, 36, 294–95.

20 Laura V. Marks, *Metropolitan Maternity: Maternal and Infant Welfare Services in Early Twentieth Century London* (Amsterdam: Rodopi, 1996), 40; Welshman, *Municipal Medicine*. For a historical discussion of civic pride, see Tom Hulme, *After the Shock City: Urban Culture and the Making of Modern Citizenship* (Woodbridge: Boydell & Brewer, 2019).

21 See George Montagu Harris, *Municipal Self-Government in Britain: A Study of the Practice of Local Government in Ten of the Larger British Cities* (London: P S King & Son, 1939); and R. B. Suthers, *Mind Your Own Business: The Case for Municipal Housekeeping* (London: Fabian Society, 1938 [1905]). The latter text was republished and revised between its initial appearance at the beginning of the century.

22 Thom, ‘Wishes, Anxieties, Play, and Gestures’; and Stewart, *Child Guidance*, 28, 63–65.

23 Thom, *ibid.*, 203. Gillian Sutherland and Stephen Sharp, ‘“The Fust Official Psychologist in the Wurrld”: Aspects of the Professionalisation of Psychology in Early Twentieth Century Britain’, *History of Science*, 1980, 15, 181–208; Wooldridge, *Measuring the Mind*, 90–103; Hall, ‘The Emergence of Clinical Psychology’.

24 Thom, ‘Wishes, Anxieties, Play, and Gestures’, 200–01.

25 Wooldridge, *Measuring the Mind*.

26 Cyril Burt, *The Young Delinquent* (London: University of London Press, 1944).

27 See Michal Shapira, *The War Inside: Psychoanalysis, Total War, and the Making of the Democratic Self in Postwar Britain* (Cambridge: Cambridge University Press, 2013), 87–111. See also the edited collection of Lowenfeld’s writings: Cathy Urwin and John Hood-Williams, eds, *Child Psychotherapy, War and the Normal Child: Selected Papers of Margaret Lowenfeld* (Eastbourne: Sussex Academic Press, 2013).

28 Hall, ‘The Emergence of Clinical Psychology’, 35.

29 Welshman, *Municipal Medicine*, 95.

30 Agatha Bowley, *Everyday Problems of the School Child* (Edinburgh: E. & S. Livingstone, 1948); Agatha Bowley, *The Problems of Family Life* (Edinburgh: E. & S. Livingstone, 1946); Agatha Bowley, *The Natural Development of the Child: A Guide for Parents, Teachers, Students and Others* (Edinburgh: E. & S. Livingstone, 1942).

31 TNA, ED 130/21, ‘The Leicester School Psychological Service Today (1964)’, typescript taken from a pamphlet, 16.

32 Thom, ‘Wishes, Anxiety, Play, and Gestures’, 214.

33 ED 130/21, ‘The Leicester School Psychological Service Today (1964)’, typescript taken from a pamphlet, 16.

34 Leicestershire, Leicester and Rutland Record Office (LLRRO), 19D59/VII/627/2, pamphlet, *The Leicester School Psychological Service, 1931–1952*, 27.

35
*Ibid*., 23–24.

36
*Ibid*., 132–42, 146–58.

37
*Ibid*., 17–18, 121–23.

38 Birmingham Archives, Heritage and Photography Service (BAHPS), BCC/1/BH/1/1/1/33, Education Committee minutes, 26 October 1934, report of Child Guidance Sub-Committee. Responsibility for the service was transferred to the Hygiene, later to become the Special Services, Sub-Committee in 1938 (see BCC/1/BH/10/1/1/28, Hygiene Sub-Committee minutes, 11 November 1938, item 8363). It should be noted that the original management of the service belonged to an obscure sub-committee established precisely for the purpose of doing so, and its records, frustratingly, do not appear to have survived.

39 See TNA, ED 137/136, N.E. Chadwick (SMO), ‘Annual Report of the School Medical Officer for the year 1944’ [Hove], 1 March 1945, 5; Thom, ‘Wishes, Anxieties, Play, and Gestures’, 213.

40 TNA, ED 137/183, letter, Brighton EO, F. Bernard Toyne to Board of Education, 6 December 1939.

41 TNA, ED 137/183, W McCartan, ‘Juvenile Care Clinic’, January 1944.

42 TNA, ED 137/182, typescript, ‘Annual Report of the Medical Inspection & Treatment of School Children of the County Borough of Brighton for the year 1947’, by SMO Rutherford Cramb and John N. Matthews, 3.

43 Stewart, ‘Child Guidance and Deinstitutionalisation’, 183.

44 Stewart, *Child Guidance*, 59, 79.

45 Harris, *The Health of the Schoolchild*, 151; Stewart, *Child Guidance*, 127–30, 132–40.

46 TNA, ED 137/135, East Grinstead Vocational and Child Guidance Clinic, ‘Report for six months. November 6^th^, 1942, to April 30^th^, 1943’. For more detail on Addis, please see Stewart, *Child Guidance*, 94–97; and Jonathan Toms, *Mental Hygiene in Twentieth-Century Britain* (Basingstoke: Palgrave Macmillan, 2013).

47 Welshman, *Municipal Medicine*, 30.

48 Stewart, *Child Guidance*, 132–43; Welshman, *Municipal Medicine*, 30; Harris, *The Health of the Schoolchild*, 176–78.

49 TNA, ED 130/31, Circular 347, 10 March 1959; *Report of the Committee on Maladjusted Children* (London: HMSO, 1955). For more on Underwood, see Stewart, *Child Guidance*, 158–65.

50 John Pickstone, ‘Psychiatry in District General Hospitals: History, Contingency and Local Innovation in the Early Years of the National Health Service’, in John Pickstone, ed., *Medical Innovations in Historical Perspective* (Basingstoke: Macmillan, 1992), 189–90.

51 BAHPS, BCC/1/BH/10/1/1/37, Hygiene Sub-Committee minutes, 9 September 1948, item 11888.

52 See LLRRO, 19D59/VII/581, Special Services sub-committee minutes, 21 July 1947—agree to joint appointment of psychiatrist with Health Committee; R/E 2/24/1, East Sussex Records Office (ESRO), R/E 2//24/1, Special Services Sub-Committee minutes; W. G. Stone, ‘Child Guidance Clinic: Report on Officers’ Conference, 2^nd^ May 1949’, 10 June 1949; BAHPS, BCC/1/BH/10/1/1/37, Hygiene Sub-Committee, 18 November 1948, item 11956; and BAHPS, BCC/1/BH/10/1/1/37, Hygiene Sub-Committee, 10 March 1949, item 12057.

53 LLRRO, 19D59/VII/582, Special Services sub-committee minutes, 6 April 1949.

54 Stewart, *Child Guidance*; Thomson, *Psychological Subjects*, 116.

55 TNA, ED 50/636, letter, Honorary Secretary of BPA (Professor Alan Moncrieff) to Underwood, 10 December 1947.

56 TNA, ED 50/636, letter, Underwood to Moncrieff, 16 December 1947.

57 Stewart, *Child Guidance*.

58 TNA, ED 50/636, Association of Education Officers, ‘Child Guidance’, enclosure with letter, F. Freeman to Sir John [?], 1 June 1949.

59 TNA, ED 50/636, minute sheet of meeting, 27 July 1949.

60 TNA, ED 50/636, letter, Soddy (NAMH) to E.W. Marris, 24 July 1948.

61 TNA, ED 50/636, interview memorandum, Ministry of Education and Ministry of Health, 30 January 1953.

62
*Ibid*.

63 See correspondence in the Summerfield files: TNA series ED 130.

64 LLRRO, 19D59/VII/581, Special Services sub-committee minutes, 19 May 1947. ‘Report of the Leicester School Psychological Service for the year ending December 31^st^, 1946’, 1.

65 LLRRO, 30D73/564, Circulars from Education Department, letter, H. S. Magnay (Director of Education) to all Primary and Secondary Schools, 20 June 1945. For more on the ‘brains trust’ and the politics of expertise in post-war Britain, see Joe Moran, ‘The Fall and Rise of the Expert’, *Critical Quarterly*, 2011, 53, 9–10.

66 LLRRO, 30D73/570, Circular, Thomas to teachers, 18 June 1954. For lectures organised by the service, see also: 19D59/VII/582, Special Services sub-committee minutes, attached typescript, ‘Report of the Leicester School Psychological Service for the year ended December 31^st^ 1953’, 1.

67 LLRRO, 19D59/VII/582, Special Services sub-committee minutes, attached typescript, ‘Report of the Leicester School Psychological Service for the year ended December 31^st^ 1953’, 2.

68 LLRRO, 19D59/VII/581, Special Services sub-committee minutes, attached typescript, ‘Annual report of the School Psychological Service for the year ending December 31^st^ 1945’, 1; and 19D59/VII/582, Special Services sub-committee minutes, attached typescript, ‘Report of the Leicester School Psychological Service for the year ended December 31^st^ 1953’, 1.

69 LLRRO, 19D59/VII/582, Special Services sub-committee minutes, attached typescript, ‘Report of the Leicester School Psychological Service for the year ended December 31^st^ 1953’, 1.

70 LLRRO, 19D59/VII/583, Special Services sub-committee minutes, attached typescript, ‘Report of the School Psychological Service for the year ended 31^st^ December 1955’, 1.

71 LLRRO, 19D59/VII/703, Special Services sub-committee minutes, attached typescript, ‘Report on the staffing of the School Psychological Service’, 5 November 1962, 1.

72 ED 130/21, ‘The Leicester School Psychological Service Today (1964)’, typescript taken from a pamphlet, 17.

73 ED 130/21, ‘The Leicester School Psychological Service Today (1964)’, typescript taken from a pamphlet, 20.

74 LLRRO, 30D73/499, memorandum, Sampson to Thomas, 19 March 1949.

75 LLRRO, 30D73/499, letter, Medical Officer of Health to Thomas, 31 March 1949.

76 TNA, ED 137/171, ‘Annual Report to the City of Birmingham Education Committee of the School Medical Officer, James R. Mitchell, M.C., M.B., ChB, DPH for the year ended 31^st^ December, 1948’, 3.

77 BAHPS, BCC/1/BH/10/1/1/37, Hygiene Sub-Committee, 15 January 1948, item 11654.

78 TNA, ED 137/171, ‘Annual Report to the City of Birmingham Education Committee of the School Medical Officer, James R Mitchell, M. C., M. B., ChB, DPH for the year ended 31^st^ December, 1948’, 24.

79 TNA, ED 137/171, ‘City of Birmingham Education Committee, School Health Service Report of the School Medical Officer Harold M. Cohen, year ended 31^st^ December 1949’, 25

80 John Walton, *The English Seaside Resort: A Social History 1750–1914* (Leicester: Leicester University Press, 1983), 66, 128–55.

81 TNA, ED 130/18, Form A2 returns, Brighton, entry for Dorothy Hammond. I am extremely grateful to one of this paper’s anonymous reviewers for the suggestion of a social science certificate as Hammond’s qualification. I have not been able to trace the exact nature of the qualification.

82 ESRO, R/E 2/24/1, Special Services Sub-Committee minutes, Dorothy Hammond, ‘Report of the Educational Psychologist upon her work during 1948’, January 1949. See also ESRO, R/E 2/24/1, Special Services Sub-Committee minutes, Dorothy Hammond, ‘Report of the Educational Psychologist upon her work during 1949’, April 1950.

83 ESRO, R/E 2/24/2, Minutes, Special Services Sub-Committee, Dorothy Hammond, ‘Report of the Educational Psychologist upon her work during 1951’, 27 January 1952.

84 ESRO R/E 2/24/2, Minutes, Special Services Sub-Committee, Dorothy Hammond, ‘Report of the Educational Psychologist upon her work during 1952’, 11 January 1953, 2.

85 ESRO, R/E 2/24/2, Minutes, Special Services Sub-Committee, Dorothy Hammond, ‘Report of the Educational Psychologist for the year 1958’, 24 February 1959.

86 Rebecca Lemov, *World as Laboratory: Experiments with Mice, Mazes and Men* (New York: Hill and Wang, 2005); Rebecca Lemov*, Database of Dreams: The Lost Quest to Catalog Humanity* (New Haven: Yale University Press, 2015). In contrast, such faith in the power of expert observation were progressively criticised in relation to children (and children’s welfare specifically), see Jennifer Crane, *Child Protection in England, 1960–2000: Expertise, Experience, and Emotion* (Basingstoke: Palgrave Macmillan, 2018).

87 Welshman, *Municipal Medicine*, 206–07.

88 TNA, ED 137/171, Harold M. Cohen, ‘School Health Service Report of the School Medical Officer’, 1951, 30.

89 TNA, ED 130/6, W. G. Stone to Aylett, 11 October 1965.

90
*Ibid*.

91 BAHPS, BCC/1/BH/1/1/1/36, Education Committee Minutes, ‘Annual Report to the City of Birmingham Education Committee of the School Medical Officer, James R. Mitchell, M. C., M. B., ChB, DPH for the year ended 31^st^ December 1938’, 42.

92 BAHPS, BCC/1/BH/1/1/1/37, Minute, 25 February 1938, report of the Finance and General Purposes Sub-Committee.

93 BAHPS, BCC/1/BH/10/1/1/28, Hygiene Sub-Committee minutes, 12 January 1939, item 8452.

94 LLRRO, 30D73/499, ‘City of Leicester Education Committee. 104, Welford Road. Schedule of alterations and additions required’.

95 LLRRO, 19D59/VII/581, Special Services sub-committee minutes, 7 February 1946. ‘Annual report of the School Psychological Service for the year ending December 31^st^ 1945’, 2.

96 TNA, ED 130/21, ‘The Leicester School Psychological Service Today (1964)’, typescript taken from a pamphlet, 16.

97 Stewart, ‘Child Guidance and Deinstitutionalisation’, 180.

98 TNA, ED 137/536, letter, H. E. Child (CEO, Dudley) to Secretary of the Ministry of Education, 9 June 1960.

99 TNA, ED 137/535, T.O.P.D. Lawson and W. Parker, ‘Annual of the Medical Officer of Health and Chief Welfare Officer … for the year 1954’, 39.

100 See the reports contained in TNA, ED 137/535.

101 TNA, ED 137/535, T.O.P.D. Lawson and W. Parker, ‘Annual of the Medical Officer of Health and Chief Welfare Officer … for the year 1956’, p. 38; ED 137/535, T.O.P.D. Lawson and W. Parker, ‘Annual of the Medical Officer of Health and Chief Welfare Officer … for the year 1958’, unpaginated typescript;

102 Sandwell Community History and Archives Service, CB/S/2/23/1/25, Education Committee, minutes, 12 December 1927, item 8031.

103 TNA, ED 137/609, letter, Chief Education Officer (Smethwick) to Secretary (Ministry of Education), 27 June 1960.

104 TNA, ED 137/608, Richard J. Dodds (PSMO, Smethwick), ‘The Health of our School Children during 1957: the annual report of the Principal School Medical Officer’, 8–9.

105 TNA, ED 137/608, Richard J. Dodds (PSMO, Smethwick), ‘School Health 1959: the annual report of the Principal School Medical Officer’, 8.

106 ‘R.J. Dodds MB, BS, FFCM, DPH’, *British Medical Journal (Clinical Research Edition)*, 1987, 295, 395.

107 TNA, ED 137/256, Hugh Paul, ‘The Health of the Children of Smethwick for the year 1948’ [SMO’s report], 10, 31.

108 See Crane, *Child Protection in England*.

109
*Psychologists in Education Services* (London: HMSO, 1968).

110 TNA, ED 130/1, memorandum, ‘Meeting to discuss the proposed working party on educational psychologists, 3^rd^ June 1964’.

111 Laura Tisdall, ‘Education, Parenting, and Concepts of Childhood in England, *c*.1945 to *c*.1979’, *Contemporary British History*, 2017, 31, 24–48.

112 TNA, ED 130/2, letter, G. D. Edwards, CEO for Cambridgeshire and Isle of Ely County Council to Aylett, 24 August 1965.

113
*Ibid*.

114 TNA, ED 130/3, Letter, [unnamed] Director of Education for Herefordshire to Aylett, 24 August 1965; ED 130/3, Letter, CEO Rutland [unnamed] to Aylett, 10 September 1965.

115 TNA, ED 130/5, Letter, Alec Clegg to Aylett, 4 September 1965. For more on Clegg, see: Peter Cunningham, ‘Innovators, Networks and Structures: Towards a Prosopography of Progressivism’, *History of Education*, 2001, 30, 433–51.

116 TNA, ED 130/5, East Sussex County Council, pamphlet, ‘Special Services. I. The School Psychological Service. II. Educationally Sub-Normal Pupils’, n.d., 1–2.

117
*Ibid*., 4.

118 TNA, ED 130/5, paper, ‘Extracts from the School Psychological Service Report’ (Wiltshire), enclosed with letter from CEO to Aylett, 8 September 1965.

119 In Birmingham, for instance, the Chief Education Officer corresponding with the committee noted this his letter was informed by the ‘personal’ views of the city’s senior educational psychologist: TNA, ED 130/6, Sir Lionel Russell to Aylett, 17 September 1965.

120 TNA, ED 50/873, minute, Aylett to Amaral, 5 December 1967.

121 TNA, ED 130/26, Child Guidance Training Centre, ‘Training course for educational psychologists (revised August 1965), 7.

122
*Ibid.*

123 TNA, ED 130/26, Tavistock Institute, ‘Memorandum on the training of educational psychologists’, 17.

124 TNA, ED 50/997, minute, Gann to Howlett, 22 July 1968.

125
*Local Authority and Allied Personal Services* (London: HMSO, 1968). According to Stewart, Seebohm made it clear that socio-economic conditions were an ‘important factor in family stress and breakdown’, which led to the need for psychiatric referral and began a shift towards local authority concern with social work: Stewart, ‘Child Guidance and Deinstitutionalisation’, 190–91. The historiography of post-war Britain has recently identified the emergence of new types of social-scientific, as much as medical, expertise as a defining feature of the period after the Second Word War, and a growing politicisation of ‘experts’: Moran, ‘The Fall and Rise of the Expert’, 11–14. Stewart notes the increasing power of the social worker within the child guidance process and the growth of social-work-based, psychosocial critiques of guidance practices as the 1950s wore on: Stewart, *Child Guidance*, 183; Hayward, *Transformation of the Psyche*. While Welshman highlights the dominant ‘trend’ of the post-war period as a ‘decline of public health and the relative rise of social work’: Welshman, *Municipal Medicine*, 241. See also: John Welshman, ‘In Search of the “Problem Family”: Public Health and Social Work in England and Wales, 1940–1970’, *Social History of Medicine*, 1996, 9, 447–65; John Welshman, *Underclass: A History of the Excluded Since 1880*, 2nd edn (London: Bloomsbury, 2013 [2006]), 79–97.

126 TNA, ED 50/873, Howlett to Rossetti, 4 March 1968; TNA, ED 50/873, Aylett to Howlett, 5 March 1968; ED 54/387, A. S. Gann, ‘Summerfield Report: Psychologists in Education Services’, 30 May 1969, 1; and ED 50/996, Gann to Hudson, 30 May 1969.

127 TNA, ED 50/1001, J.A. Hudson to Secretary, 22 February 1971.

128 TNA, ED 50/1001, Hudson to Secretary, 1 January 1974.

129 TNA, ED 50/1001, W. D. Pile to Hudson, 4 March 1971. Visualisation by civil servants was also an issue in relation to the NHS, a point for which I am grateful to Roberta Bivins, ‘Charting the NHS’, *People’s History of the NHS* website, 8 June 2018 [accessed at https://peopleshistorynhs.org/charting-the-nhs/?highlight=visual on 13 March 2019].

130 TNA, ED 50/1004, Dudman (legal advisor) to Crawford, 11 March 1970; see also TNA, ED 50/1004, Dudman to Crawford, 7 April 1970.

131 TNA, ED 50/1004, Didsbury to Farrant, 29 April 1970; TNA, ED 50/1004, Crawford to Carswell, 9 April 1970; TNA, ED 50/1004, ‘Future responsibility for Child Guidance Services: note of inter-departmental meeting held at Alexander Fleming House, 27 May 1970’. See also TNA, ED 50/1004, Mayer to Crawford, 30 June 1970.

132 Eliot Freidson, *Profession of Medicine: A Study of the Sociology of Applied Knowledge* (Chicago: University of Chicago Press, 1970); Toby Gelfand, ‘A “Monarchical Profession” in the Old Regime: Surgeons, Ordinary Practitioners, and Medical Professionalization in Eighteenth-Century France’, in Gerald L. Geison, ed., *Professions and the French State, 1700–1900* (Philadelphia: University of Pennsylvania Press, 1984), 149–80; Matthew Ramsey, ‘The Politics of Professional Monopoly in Nineteenth-Century Medicine: The French Model and its Rivals’, in Geison, ed., *Professions and the French State*, 225–305; Magali Sarfatti Larson, *The Rise of the Professionalism: A Sociological Analysis* (Berkeley: University of California Press, 1977).

133 Hayward, *The Transformation of the Psyche*. For consumerism, education and health, see Jane Hand, ‘Marketing Margarine: Advertising, Gender and Healthy Eating in Britain from 1954–*c*.2000’, *Contemporary British History*, 2017, 31, 477–500; Mark Jackson and Martin Moore, eds, *Balancing the Self: Medicine, Politics and the Regulation of Health in the Twentieth Century* (Manchester: Manchester University Press, 2018); Alex Mold, *Making the Patient-Consumer: Patient Organisations and Health Consumerism in Britain* (Manchester: Manchester University Press, 2015); Alex Mold, ‘“Everybody Likes a Drink. Nobody Likes a Drunk”: Alcohol, Health Education and the Public in 1970s Britain’, *Social History of Medicine*, 2016, 30, 612–36.

